# Supplementary material for: The Role of Native T1 and T2 Mapping Times in Identifying PD-L1 Expression and the Histological Subtype of NSCLCs
Source: Cancers (Basel). 2023 Jun 20;15(12):3252. doi: 10.3390/cancers15123252 (PMC10296658; doi:10.3390/cancers15123252)
Supplement: Supplementary file 1 [file cancers-15-03252-s001.zip › cancers-2410764-supplementary.pdf]

SUPPLEMENTARY MATERIAL

Patient ID	T1 core	T1 mean	T1 micro-env. 3 mm	T1 micro-env. 6 mm	T1 periphery	T1 lung	T2 core	T2 mean	T2 micro-env 3mm	T2 micro-env 6mm	T2 periphery	T2 lung	Histotype	PD-L1 expression
Patient 1							133	86,57	77	80	68	207	SCC	90%
Patient 2	1184	1136	839	785	1086	732	80	87,5	203	216	88	250	S.D.	90%
Patient 3	1024	1128,5	1341	852	1059	921	83	93,8	207	231	103	256	S.D.	<1%
Patient 4	1175	1116,75			1148	962	77	87			79	163	S.D.	35%
Patient 5	1033	887,6	853	814	1056	872	91	91	198	203	95	255	S.D.	<1%
Patient 6	1012	1097,5	920	919	1111	900	84	88	194	213	94	212	SCC	80%
Patient 7	1223	1321,6	977	999	1223	811	132	156,5	225	233	137	229	S.D.	90%
Patient 8	1152	1096,5	925	952	1172	895	108	87	109	116	89	198	ADK	5%
Patient 9	1031	1165	992	954	1124	805	148	176	234	239	143	257	S.D.	<1%
Patient 10	1152	1229,2	937	814	1152	732	93	92,9	157	150	82	241	SCC	60%
Patient 11		928					78	79,3	127	136	88	221	SCC	<1%
Patient 12	1051	1272	877	830	1154	710	125	144,6	216	219	152	257	ADK	90%
Patient 13	1132	1139	1020	1031	1169	927	85	93	147	158	90	231	ADK	5%
Patient 14	903	886	893	879	835	869	79	75,7	213	214	103	198	SCC	80%
Patient 15	1159	1145	1128	947	1229	819							ADK	<1%
Patient 16	1132	1197,5	844	850	1175	828	89	90	210	235	105	257	ADK	5%
Patient 17	1036	993,5	995	1021	1029	912	100	108	159	177	103	254	ADK	<1%

Patient 18	1179	1157	941	951	1141	919	82	95	139	151	93	206	ADK	<1%
Patient 19	1184	1174	984	982	1137	872	83	87	89	91	80	224	SCC	30%
Patient 20	1079	1142	949	919	1133	933	130	145	229	231	150	257	SCC	<1%
Patient 21	1295	1206	1061	967	1164	912	186	150	224	236	176	255	ADK	>1%
Patient 22	1137	1167	1018	985	1117	912	103	112,25	203	218	116	249	ADK	<1%
Patient 23							92	105,4	203	207	109	238	ADK	<1%
Patient 24	1193	1214	1079	1069	1347	919	91	84,4	84	83	86	180	ADK	<1%
Patient 25	1178	1171,5	1007	991	1199	814	115	121,75	180	194	130	257	ADK	1-49%
Patient 26		909,5					99	105,4	187	181	96	234	S.D.	>50%
Patient 27	1197	1271,5	845	877	1270	967	65	105,8	201	210	68	238	ADK	<1%
Patient 28	689	745	828	826	760	907	65	80	178	183	81	249	SCC	10%
Patient 29	1034	1033	976	956	1017	971	102	112	205	205	99	251	SCC	1-49%
Patient 30							156	155	246	249	159	257	SCC	10%
Patient 31		1123,5						93,2					S.D.	
Patient 32	989	1061,5	939	951	1087	896	104	110,2	219	220	134	256	SCC	
Patient 33	1141	1175,75	1121	1128	1208	957	108	110,6	137	142	97	255	S.D.	

Table s1. Table of 33 patients enrolled in the histotype study, including the 30 available PD-L1 values. The PD-L1 expression is reported in its original form, in our study it has been discretized with a 1% threshold. Unit of measure of T1 and T2 mapping time is ms.
